# A Synthetic Literature Review on the Management of Emerging Treatment Resistance in First Episode Psychosis: Can We Move towards Precision Intervention and Individualised Care?

**DOI:** 10.3390/medicina56120638

**Published:** 2020-11-24

**Authors:** Siân Lowri Griffiths, Max Birchwood

**Affiliations:** 1 Institute for Mental Health, University of Birmingham, Birmingham B15 2TT, UK; 2 Warwick Medical School, University of Warwick, Coventry CV4 7AL, UK; M.J.Birchwood@warwick.ac.uk

**Keywords:** first episode psychosis, treatment resistance, psychosis, clozapine, CBT, early intervention

## Abstract

Treatment resistance is prevalent in early intervention in psychosis services, and causes a significant burden for the individual. A wide range of variables are shown to contribute to treatment resistance in first episode psychosis (FEP). Heterogeneity in illness course and the complex, multidimensional nature of the concept of recovery calls for an evidence base to better inform practice at an individual level. Current gold standard treatments, adopting a ‘one-size fits all’ approach, may not be addressing the needs of many individuals. This following review will provide an update and critical appraisal of current clinical practices and methodological approaches for understanding, identifying, and managing early treatment resistance in early psychosis. Potential new treatments along with new avenues for research will be discussed. Finally, we will discuss and critique the application and translation of machine learning approaches to aid progression in this area. The move towards ‘big data’ and machine learning holds some prospect for stratifying intervention-based subgroups of individuals. Moving forward, better recognition of early treatment resistance is needed, along with greater sophistication and precision in predicting outcomes, so that effective evidence-based treatments can be appropriately tailored to the individual. Understanding the antecedents and the early trajectory of one’s illness may also be key to understanding the factors that drive illness course.

## 1. Introduction

Treatment resistance in psychosis has traditionally been dominated by the Kraepelinian view of chronic disorder associated with enduring impairment [1]. In reality, the outlook is positive for many individuals with first episode psychosis (FEP; please see Table 1 for a list of abbreviations, but there remains a subgroup of individuals who do not achieve a symptomatic and/or functional recovery, despite receiving specialised care under an early intervention service (EIS) [2,3]. Two longitudinal studies have shown that of individuals who were identified as treatment resistant, over 70% were treatment resistant from illness onset [4,5]. Indeed, initial response to treatment is one of the strongest predictors of longer-term outcomes in those with early psychosis [6,7]. The prolongation of symptoms and continued disruption to an individual’s peer relationships and work and school performance at this time is likely to have a profound negative impact on their future [8]. It is therefore imperative that these individuals are identified as early as possible in their illness trajectory and given appropriate stratified interventions to improve outcomes and prevent further decline. 

## 2. Defining Treatment Resistance

The lack of consensus over a clear definition of treatment resistance and validated instruments to assess the concept remains problematic for research in this area [9,10]. Treatment resistance is often conceptualized by the persistence of unremitted positive symptoms, despite the sequential use of at least two antipsychotic medications at a therapeutic dose, for a minimum duration of six weeks [4,11].

However, this definition disregards other symptoms which are shown to influence outcomes in psychosis, such as cognitive impairment and negative symptoms, which tend to be unresponsive to standard treatments [12,13,14]. The lack of broadness in the definition of treatment resistance has also meant that other key outcome domains, such as one’s social and role functioning and quality of life, are overlooked [15]. Finally, the term itself may be conceived as stigmatizing, as it places onus on the individual for ‘not responding adequately’ or ‘failing’ their treatment, which may add to the increased shame and stigma that an individual may already experience as a result of receiving their diagnosis [16]. 

An alternative definition that has been proposed is the concept of ‘incomplete recovery’, which considers persisting impairment in psychosocial and functional domains, despite intervention with evidence-based psychosocial and pharmacological treatments; the term also reflects the potential for improved therapeutic outcomes [10,17]. Within this concept, the lack of recovery following adequate treatment is distinguished from non-adherence to treatment; each may contribute to incomplete recovery and should be identified and addressed early to promote recovery in FEP [9,10].

## 3. Prevalence and Predictors of Treatment Resistance and Incomplete Recovery

The rates of treatment resistance and incomplete recovery vary widely in FEP. This disparity is likely a result of studies adopting different criteria to define treatment resistance. The inclusion of participants with affective and non-affective psychosis is also likely to add to this heterogeneity, given that outcomes tend to be more favorable in those with prominent affective trajectories [18,19]. Recent publications in longitudinal FEP cohorts report rates between 22–34% for individuals who are resistant to antipsychotic medication [5,20,21]. Findings from systematic reviews on relapse and incomplete symptomatic recovery in FEP again show variation in the rates reported (19–89%), but notably, the risk of relapse is significantly reduced by sustained antipsychotic therapy [3,22,23,24]. Finally, incomplete recovery within social and vocational domains are shown to vary between 46% and 86% in FEP [3,25]. These findings highlight that current evidenced-based treatments and services are not adequately addressing the needs of all its service users.

There is also complexity in predicting outcomes in FEP; incomplete recovery appears to be multidimensionally determined and impacts separable domains of recovery [1,17]. Variables shown to contribute to incomplete recovery include: long delay in untreated psychosis (DUP), younger age at onset of psychosis, poorer premorbid adjustment, cognitive impairment, negative symptoms, affective comorbidity, non-adherence and disengagement with treatment, male gender, and initial response to treatment [4,5,14,26,27,28,29,30,31]. It is likely that each individual will have their own unique combination that will determine their outcome, which makes predictions at the individual level challenging, particularly in the early stages of psychosis where illness trajectories are forming and the clinical picture is still emerging [1]. 

It is also likely that illness trajectories are long-standing, and impairment pre-dates formal illness onset [32]. Supporting this view is the dimensional approach to illness whereby the heterogeneity in early psychosis is characterized by differing illness subtypes, rooted early in development [33,34]. Indeed, early cognitive and neurodevelopmental impairment has been associated with psychosis liability and core schizophrenia [15,35], and it is hypothesised that aberrations in the neurodevelopmental process, linked to cognitive deficits, lie at the heart of early and enduring impairment in psychosis [15]. Such an approach may have utility for subtyping individuals on their early course or premorbid features, potentially aiding in the parsing of clinical heterogeneity [33].

There are also a number of established social antecedents in the development of psychosis; an accumulation of these risk and protective factors over time may not only increase someone’s risk of psychosis, but may continue to operate within the process of recovery [1]. It is established that social gradients are heavily implicated in the development of psychosis, such as: urbanicity, social marginalization and fragmentation, ethnic minority status, and childhood adversity [36,37]. These factors are likely to lead to a vicious cycle of disadvantage, which, if not addressed, will continue to drive enduring impairment [38]. For example, hostile and critical family environments are associated with relapse and depression in psychosis, and changing these environments via family interventions are indeed shown to be effective at reducing relapse rates [39]. More recently, the concept of urban remediation in psychosis has been proposed as a new recovery-oriented strategy to manage urban stress, but at present, this remains a goal for future research [40].

From the evidence presented above, there appears to a gap in the knowledge base for understanding the evolutionary continuity between alterations in neurodevelopmental process and exposure to stressful life events, with later onset of psychosis [34]. Longitudinal prospective studies in children and young people are essential to improve our understanding of neurodevelopmental markers in children and young people with varying levels of psychosis risk, and how these might be linked to long term prognoses.

## 4. Current Approaches for Managing Emerging Treatment Resistance in Early Psychosis

The first 3–5 years following illness onset, including the period of untreated psychosis, represents a ‘critical period’ of illness progression [7,41] It is during this time in which interventions are likely to have their greatest impact [41].

EIS provides specialist assertive outreach-style care during the ‘critical period’, and are effective at improving a number of clinical and functional outcomes for young people with FEP [42]. However, there appears to be a group of individuals whose psychosis remains ‘unresponsive’ to standard high quality EIS care, embodying National Institute for Clinical Excellence (NICE) approved psychosocial and pharmacological interventions; strongly suggesting that earlier, targeted interventions are urgently needed to allow such individuals to maximise their life chances [3,21].

A recent publication by Drake and colleagues [27] demonstrated the importance of having such timely interventions. In their longitudinal modelling study, they demonstrated a curvilinear relationship between DUP with symptom severity and treatment response, meaning that symptoms become more refractory with a longer DUP, but this response was more rapid at first and then plateaued [27]. These recent findings place even greater emphasis on providing individuals with prompt access to a range of interventions ideally within the first few weeks of illness onset [27]. 

However, despite the implementation of EIS leading to a significant reduction DUP, a proportion of individuals continue to have DUPs exceeding 6 months [43,44]. Furthermore, whilst earlier studies provide evidence that intervention programmes can be successful at reducing DUP, for example, the Treatment and Intervention in Psychosis Study (TIPS), where DUP was reduced from 16 to five weeks, a recent systematic and meta-analytic review of 16 studies did not find any conclusive summary evidence for controlled interventions in reducing DUP [45,46]. 

Reducing DUP, particularly in areas where DUPs are consideringly long, should remain a priority to improve outcomes. Large scale collaborative studies with rigorous study designs and robust assessments of DUP are needed to inform more effective interventions [46].

Another important finding by Drake and colleagues was that DUP was not linked to any symptoms at presentation, except depression [27]. It is established that depression in psychosis is associated with a range of poor outcomes, but yet there is a lack of large-scale controlled trials investigating the effectiveness of adjunctive antidepressants, or cognitive behavioural therapy (CBT), to target depression within psychosis [26,47,48]. This calls for more effective recognition and management of depression to maximise recovery within EIS. 

NICE guidelines in the UK advocate for antipsychotic medication and CBT as first line treatments for all individuals with psychosis [11]. Clozapine is the only evidence-based treatment for refractory psychosis, and current guidelines recommend commencement after two unsuccessful trials of standard antipsychotics [11,49]. A recently published study investigating the pathways to prescribing clozapine for treatment resistance within UK EIS centres, showed a marked delay in clozapine prescription for those who were eligible [21]. A clear stasis in treatment was evident for these individuals, where the majority either remained on the same medication despite persisting symptoms, or they continued to be placed on other antipsychotics which were also unsuccessful [21]. 

Given the evidence of the superior efficacy of clozapine in reducing suicide risk, improving symptomatic and functional recovery, and reducing mortality rates, this may reflect a missed opportunity to influence recovery during the significant ‘critical period’ [21,50,51].Ongoing education of the benefits of clozapine and emphasis on the national standards for commencement of clozapine in the community is perhaps needed to improve uptake on clozapine for those who are eligible [22]. But despite its superiority in treating refractory symptoms, it must be acknowledged that 30–40% of individuals will still show an insufficient response to clozapine, and others are unable to tolerate the medication; the move toward community commencement of clozapine would also require national standards on clozapine discontinuation for those who are unable to tolerate [52,53].

Conventional CBT for psychosis is also less likely to be effective for subgroups of individuals with particularly complex illness presentations [52,54,55], and there is considerable heterogeneity in response to psychosocial interventions in psychosis [56]. Baseline factors are shown to contribute a large amount of this variance. For example, cognitive impairments are shown to have a rate-limiting impact on treatment progress [57,58].

Encouragingly, a recent randomised controlled trial provides an example of a tailored approach to those who are treatment resistant within early psychosis. A specialised social CBT, namely social recovery therapy (SRT), is shown to be effective at increasing structured activity in a sizeable group of young people whose severe social disability had proved unresponsive to standard EIS [54]. Potentially, individuals who are likely on a pathway to treatment resistance may receive SRT earlier in their illness trajectory to prevent their disability from becoming entrenched.

The benefits of such an intervention may also potentially extend to individuals who are disengaged with their treatment; the basis of SRT is to motivate individuals who are perhaps ambivalent about change, whilst also addressing any underlying blocks to change, or in this instance, interventions such as SRT may be helpful to address any underlying reasons for non-adherence to treatment [54].

Finally, there is further scope for the refinement of the SRT to ensure that the therapy is being delivered appropriately, such as greater precision in the identification of individuals who are less likely to benefit from such interventions, and a greater understanding of the mechanistic markers of change to inform the process of early treatment stratification [59].

## 5. A Move towards Precision Intervention and Stratified Treatment Approaches in Early Psychosis

The move towards ‘big data’ has meant that novel statistical approaches may allow for the delineation of the complexity and multiplicity within mental illnesses [1]. Mathematical modelling techniques, such as machine learning, apply computer algorithms to test complex models, and have the potential to inform personalized diagnostic tools to help clinicians guide treatment at an individual level [60]. 

To-date, classical inferential approaches have dominated the field, but are becoming increasingly scrutinized for their lack of reproducibility [61]. Whilst the arbitrary p-value cut-off of 0.05 may produce significant findings, it does not accurately account for replicability and generalizability [61]. For example, a replication rate of around 11% has been estimated for preclinical studies [62], and for high dimensional data, there is a greater probability of having false-positive findings; neuroimaging analyses have a 70% false-positive rate even after correction for multi-comparisons [61,63]. This suggests that a great deal of significant findings are perhaps reflecting the noise or idiosyncrasy of the data, and less likely to generalize [61].

With machine learning, one can test the accuracy and generalizability of models through a process of cross-validation, which may include the models being independently validated across different study sites [61]. The performance of the model can then be externally validated on a sample from a different study, giving an indication of the model’s overall generalizability. The final stage in the generalizability assessment is prospective validation, which involves the application of an existing model to a new individual to make a generalizable prediction [61].

Data from a range of modalities (e.g., biological, neuroimaging, genetic, and clinical data), can also be fused together to capture different illness phenotypes and mechanistic markers, potentially leading to a more objective nosology and improved prognostic certainty [60,64].

There is emerging evidence demonstrating the prognostic capabilities of machine learning within mental health, with accuracies of above 70% being achieved so far across a growing number of studies [65]. This has been extended to symptomatic and functional remission in early psychosis [3,60]. However, so far, multimodal approaches have not yet incorporated environmental and societal factors, which are associated in poor outcomes and strongly implicated in the pathway to psychosis [66].

Using multimodal data, transdiagnostic features can be decomposed further; for example, neuroimaging data can be trained using unbiased or unsupervised machine learning methods to divide data into subgroups [67]. This could reveal which transdiagnostic features are intrinsic to early psychosis, and has the potential to inform adjunctive treatments that may lead to better outcomes [67].

Finally, the way in which treatments are currently evaluated are focused on finding the most efficacious treatment for the average patient [68]. However, given the heterogeneity in response profiles, machine learning may have utility within clinical trial designs to identify individuals who are more likely to respond to intervention, and this could ensure that costly interventions (for both health service and service user), are delivered to those who are more likely to benefit.

Using neuroimaging data, machine learning has so far been able to predict treatment response to intervention within anxiety disorders with up to 80% accuracy [68,69], and similarly with high accuracy for depression following treatment with CBT and antidepressant medication [64,70]. As well as holding promise for stratifying treatments, the identification of markers of non-response may lead to the development of more suitable interventions.

## 6. The Application of Machine Learning and Model Translation

The growing body of evidence is beginning to demonstrate the clinical application of machine learning to everyday practice, with the potential to inform more objective diagnoses, illness prognoses, and stratification of interventions [64]. Implementation into everyday practice may take several forms, for example, the release of publicly available algorithms, and the integration of clinical information from within health systems and personal monitoring devices such as smart phones [71,72]. However, as the field moves towards translation, it is important to acknowledge the limitations and ethical implications.

### 6.1. Practical Considerations

Firstly, it is important to consider the real-world practicalities of implementing machine learning into everyday practice. Machine learning often draws on data from a range of modalities (e.g., task-based imaging), and such technologies may not be available on a large scale [64]. Additionally, machine learning techniques are often complex to implement and interpret, and would require prior knowledge and skills of the already time-pressured clinician.

### 6.2. Sample Representativeness and Diversity

It is imperative that samples in which the models are tested, accurately represent the individuals whose treatments will be informed by the prediction models in clinical practice. This requires large and diverse samples that represent real-world heterogeneity [65]. However, studies investigating neuroimaging biomarkers demonstrate a decrease in accuracy with larger samples (N => 150), which might actually result from sample heterogeneity [71]. Smaller, more homogenous samples might be able to achieve higher prediction accuracies, but this might compromise the model’s generalizability, and thus its translation to clinical practice [71]. 

Epidemiological studies demonstrate the importance of sample diversity. There is higher incidence of psychosis in black minority ethnic groups, and these individuals are also much more likely to have poor outcomes across a range of domains [72,73,74]. Yet, minority ethnic groups are often underrepresented in clinical research studies, and subsequently, machine learning prediction models may not generalize to those who are at risk of the poorest outcomes [61,64]. The AESOP-10 cohort study investigated the disparity in illness outcomes between black minority ethnic and white British patients, and showed no difference in baseline factors such as demographic, clinical, neurodevelopmental, and substance misuse. Social outcomes were also largely independent of clinical course [73]. Though it’s a tentative finding, baseline social disadvantage and isolation contributed to persistence of symptoms and social outcomes for the black African and black Caribbean individuals [73].

These findings are important for the translation of prediction models. It demonstrates that the complexity of illness outcomes extends beyond the clinical features, and may mirror the inequity in our health systems and social structures for these marginalized groups [73]. Environmental factors such as urbanicity, pervasive disadvantage, marginalization, and adversity, which lead to increased rates of psychosis, are also likely to continue to operate within the process of recovery [36,37]. To improve outcomes, there is an apparent need to understand how these predisposing factors are continuing to drive illness course.

Recent attempts have been made to provide an aggregate measure of environmental risk to psychosis [66]. The Maudsley Environmental Risk Score – which is a cumulative load of environmental risk factors – might be an important addition when developing prediction models within psychosis, and may yield more generalizable findings [66].

### 6.3. Ethical Considerations

There are a number of ethical implications associated with the integration of machine learning into routine health care. For example, the prognostic potential of machine learning means that the individual might be told of future relapses, which may cause unnecessary distress [64].

Furthermore, data gathered from personal devices introduces further concerns around compulsion in relation to data surveillance, and overlooks the autonomy of the individual, potentially creating further power inequalities in the mental health system [75].

Finally, there are ethical concerns regarding the transparency of the machine learning algorithms [64]. The model’s operations and decisions should be scrutinized, and clinical decisions not left to the machine alone [75]. Rather, it should be used as a system to help guide the clinician’s decisions, without needing to remove the human aspect of care, nor needing to replace the individual’s narrative.

## 7. Conclusions

The complexity and heterogeneity in early illness course makes predictions at an individual level challenging, and many continue to show non-response to conventional, NICE-embodied interventions. The application of machine modelling shows some promise in being able to delineate this complexity and potentially improve prognostic certainty. It may also allow for a targeted treatment approach to guide interventions for whom they will most benefit.

However, just as the factors that contribute to psychosis and illness outcomes are dynamic and multidimensional, it is likely that the same will be true for new treatments to promote recovery for those with complex presentations. Understanding the predisposing factors leading to one’s illness may also be key to promoting and sustaining one’s recovery in the long term.

## Figures and Tables

**Table 1:** Abbreviation Legend.

Abbreviated Term	Full Term
FEP	First Episode Psychosis
EIS	Early Intervention in Psychosis
DUP	Delay in untreated psychosis
NICE	National Institute for Clinical Excellence
CBT	Cognitive Behavioural Therapy
SRT	Social Recovery Therapy
